# Survey on the Presence of Viruses of Economic and Zoonotic Importance in Avifauna in Northern Italy

**DOI:** 10.3390/microorganisms9091957

**Published:** 2021-09-15

**Authors:** Tiziana Trogu, Sabrina Canziani, Sara Salvato, Clara Tolini, Guido Grilli, Mario Chiari, Marco Farioli, Loris Alborali, Alessandra Gaffuri, Giovanni Sala, Alessandro Bianchi, Carlo Rosignoli, Paola Prati, Matteo Gradassi, Enrica Sozzi, Davide Lelli, Antonio Lavazza, Ana Moreno

**Affiliations:** 1 Istituto Zooprofilattico Sperimentale della Lombardia e dell’Emilia Romagna “Bruno Ubertini” (IZSLER), Via Antonio Bianchi 7/9, 25124 Brescia, Italy; tiziana.trogu@gmail.com (T.T.); sara.salvato@izsler.it (S.S.); clara.tolini@izsler.it (C.T.); giovanni.alborali@izsler.it (L.A.); alessandra.gaffuri@izsler.it (A.G.); giovanni.sala@izsler.it (G.S.); alessandro.bianchi@izsler.it (A.B.); carlo.rosignoli@izsler.it (C.R.); paola.prati@izsler.it (P.P.); matteo.gradassi@izsler.it (M.G.); enrica.sozzi@izsler.it (E.S.); davide.lelli@izsler.it (D.L.); antonio.lavazza@izsler.it (A.L.); anamaria.morenomartin@izsler.it (A.M.); 2 Dipartimento di Medicina Veterinaria, Università degli Studi di Milano, Via dell’Università 6, 26900 Lodi, Italy; guido.grilli@unimi.it; 3 Direzione Generale Welfare, Regional Health Authority of Lombardy, 20124 Milan, Italy; Mario_Chiari@regione.lombardia.it (M.C.); Marco_Farioli@regione.lombardia.it (M.F.)

**Keywords:** wild birds, avian influenza, West Nile virus, Usutu virus, Newcastle disease, Italy

## Abstract

Wild birds play an important role in the circulation and spread of pathogens that are potentially zoonotic or of high economic impact on zootechnical production. They include, for example, West Nile virus (WNV), Usutu virus (USUV), avian influenza virus (AIV), and Newcastle disease virus (NDV), which, despite having mostly an asymptomatic course in wild birds, have a strong impact on public health and zootechnical production. This study investigated the presence of these viruses in several wild bird species from North Italy during the biennium 2019–2020. Wild birds derived from 76 different species belonging to 20 orders. Out of 679 birds, 27 were positive for WNV (lineage 2) with a prevalence of 4%; all birds were negative for USUV; one gull was positive for H13N6 influenza virus, and 12 samples were positive for NDV with a prevalence of 2%. Despite the low prevalence observed, the analyses performed on these species provide further data, allowing a better understanding of the diffusion and evolution of diseases of both economic and zoonotic importance.

## 1. Introduction

Attention to wildlife monitoring has always been very high in relation to public health, especially considering that more than 70% of the emerging zoonotic diseases derive precisely from free ranging wildlife [1]. The recent identification of SARS-CoV-2 and its subsequent pandemic diffusion have sparked an increased interest in these issues and the close relations involving animals, the environment, and public health.

Among wild species, birds play a crucial role in the mechanisms of distribution, persistence, and evolution of certain pathogens, potentially zoonotic, with an economic impact on poultry farming. They are known to be reservoirs for several bacteria, such as *Salmonella* spp. [2], drug-resistant organisms, parasites, or viruses. In this regard, avian influenza virus (AIV), Newcastle disease virus (NDV), and vector-borne diseases such as West Nile (WNV) and Usutu virus (USUV), could represent important zoonotic agents and/or have a high economic impact on zootechnical production.

Avian influenza is caused by the influenza virus type A, belonging to the family Orthomyxoviridae. Wild waterbirds from the order Anseriformes (mainly ducks, swans, and geese) and Charadriiformes (gulls and terns) are natural reservoirs of low pathogenic influenza (LPAI) [3]. In these species, the virus LPAI usually circulates asymptomatically, whereas poultry can show mild to severe clinical forms. However, the possible evolution of the H5 and H7 subtypes into highly pathogenic influenza virus (HPAI) is the most relevant aspect, since they can cause high mortality in both wild species and domestic poultry, with severe economic consequences. In addition, reassortment between subtypes poses a risk for immune escape in these and in new hosts susceptible to infection, such as swine, equids, and humans [4], raising potential pandemic threats [5].

ND, which is of lesser zoonotic importance [6], is still a major problem in poultry farming. NDV (*Orthoavulavirus*-1), or avian paramyxovirus-1 (APMV-1) belongs to the family Paramyxoviridae and genus *Avulavirus*. It represents a major limiting factor for poultry production in many developing countries, and even in developed countries, despite increased biosecurity measures and vaccination, it causes some sporadic outbreaks. The clinical signs of infected birds vary depending on the host species, immune status, and age, as well as the virulence and dose of the virus; thus, it could be difficult to recognise the disease [7]. NDV has been isolated from numerous species of wild birds, considered natural reservoirs of lentogenic strains, and, occasionally, velogenic strains [8,9,10]. Most strains isolated from wild birds would appear to be non-pathogenic to poultry; however, outbreaks of virulent NDV in poultry originating from avirulent strains from natural reservoirs have been reported [11].

WNV and USUV are closely related arthropod-borne viruses (genus *Flavivirus*; family *Flaviviridae*). These agents are transmitted by mosquitoes, and the infections they cause represent important public and animal health concerns because of their wide geographical diffusion and the broad range of potentially affected hosts, particularly birds and mammals, including equids and humans. Mammals are considered accidental or dead-end hosts, rarely developing sufficient viremia to re-infect feeding mosquitoes [12]. Instead, wild birds are considered the main vertebrate reservoir hosts of WNV and USUV and the main amplifying hosts of the viruses in nature [13,14]. Indeed, they are able to develop a strong and long-term viremia and are capable of infecting bird-biting mosquitoes [15]. European epidemiological data show a severe increase in human cases that have occurred in the last ten years, raising the focus on surveillance of these arboviruses through combined monitoring of vectors, wild birds, humans, and the environment.

Considering the characteristics of the viruses described, wild birds may have a predominant role in the epidemiology of diseases. In particular, the ability to migrate over long distances allows them to play a role in the amplification and circulation of viruses [16], thus creating the potential for the establishment of new endemic foci of disease along migration routes from endemic areas. Indeed, infected migratory wild birds have been suspected of spreading HPAI virus subtype H5N1 from central Asia to Eastern Europe through migratory flyways in 2005 [17], and USUV is thought to have been introduced in Europe during bird migration, at different times starting in the 1950s [18]. In addition, for some agents, wild birds can also act as a mechanical vector passively carrying infected insects or parasites, even if birds are not a competent reservoir for that particular infection [19]. Wetlands along migratory routes allow the concentration of many birds of different species, thus promoting intra- and interspecific transmission and playing an important role in local and long-distance viral dispersal [20,21].

In this epidemiological context, monitoring of wild bird populations provides information about pathogen spread and virus circulation, allowing the early detection of possible new outbreaks. However, observation of clinical symptoms and signs is somewhat problematic in wild birds, considering the difficulty in capturing them individually for examination. Often, only the phenomena of high mortality allow the detection of the circulation of certain pathogens [22]. Therefore, these aspects, along with the severe impact of the described diseases, highlight the importance of systematic surveillance of the avifauna in the presence of viral agents.

In Italy, surveillance plans for wild target species for avian influenza [23] and arbovirosis [24] are active and regularly performed. Therefore, this study aimed to investigate the presence of the above-mentioned viruses in several species of wild birds, derived from passive surveillance within the Lombardy (Northern Italy) regional plan for wildlife monitoring.

## 2. Materials and Methods

The samples examined were gathered in the framework of the wild bird monitoring plan that took place in the Lombardy region (Northern Italy) during the years 2019–2020. Wild bird carcasses from the wildlife rehabilitation centres (CRAS) and regional veterinary units of Lombardy were committed to the Istituto Zooprofilattico Sperimentale della Lombardia e dell’Emilia Romagna (IZSLER). In most cases, subjects required immediate euthanasia, sometimes they were already dead when conferred, and, in the remaining cases, the average time spent in the CRAS was 1–2 days before death.

**Figure 1:**
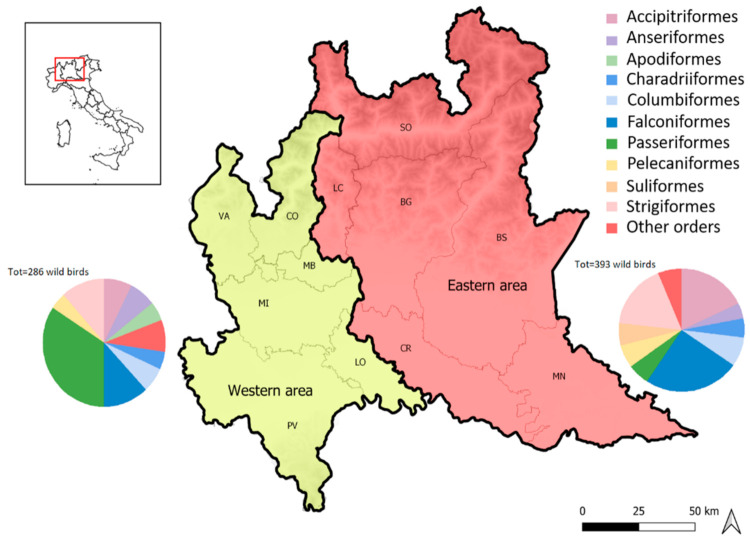
Representation of study areas (western and eastern). In the graphs, the most sampled orders for the two different areas are represented.

Two main areas of Lombardy, from which the largest number of samples have been sent, could be identified: (i) an eastern area that includes samples from Brescia, Bergamo, and Lecco provinces, in particular from Parco Adamello and Val Predina CRAS and (ii) a western area that includes Milano, Pavia, Monza, Varese, and Como provinces, referring to La Fagiana and Vanzago CRAS. From the eastern area, 393 samples were conferred, while 286 samples were from the western area, for a total of 679 wild birds analysed from 76 different species belonging to 20 orders (Figure 1).

The greatest number of species belonged to the orders Strigiformes, Anseriformes, Accipitriformes, Passeriformes, Charadriiformes, and Falconiformes (for more details, and to consult the main results, see Table S1 in the Supplementary Materials).

For each wild bird, organs such as brain, lungs, heart, kidneys, spleen, and intestine were homogenised and subsequently centrifuged at 3750× *g* for 15 min to favour the precipitation of cells and proteins that could interfere in later stages. Total viral RNA was extracted from 100 µL of homogenised samples using Qiagen BioSprint 96 One-For-All Vet 100 (Qiagen, Hilden, Germany) and stored at −80 °C or immediately used. The internal control exogenous RNA template and carrier RNA were added to each sample prior to extraction. RNA from all samples was subjected to a panel of real-time RT-PCR to detect West Nile virus, Usutu virus, influenza A virus, and Newcastle virus. For any reaction, 5 μL of RNA was added to the master mix PCR, and positive and negative controls (no template) of amplification were included. The primers and probes used for the different PCRs are listed in Table 1.

Real-time RT-PCR for influenza, WNV, and USUV was performed using the QuantiFast Pathogen RT-PCR with IC Kit (Qiagen, Hilden, Germany), while for the detection of NDV and WNV Lineage 1–2, the QuantiTect Probe RT-PCR Kit (Qiagen, Hilden, Germany).

The amplification reaction for the detection of WNV [25] and USUV [28] was conducted with a thermal profile of 50 °C for 20 min, 95 °C for 5 min, and cycles 45 of 95 °C for 15 s and 60 °C for 30 s. West Nile positive samples were subjected to real-time RT-PCR to distinguish lineage 1 or 2 [26,27]. PCR was carried out using QuantiTect probe RT-PCR Kit (Qiagen, Hilden, Germany) in which, into each sample were added a pair of primers forward and reverse and 2 different probes: one specific for lineage 1 and the other specific for lineage 2. The thermal profile used was 50 °C for 30 min, 95 °C for 15 min, and 45 cycles at 95 °C for 15 s and 60 °C for 1 min.

For the detection of AIV [29], the amplification reaction was conducted with the same thermal profile used for WNV and USUV, but for 40 cycles instead of 45. Influenza-A-positive samples were subjected to two typing real-time RT-PCRs to define the subtype H5 or H7 [30]. Two reaction mixes were set up with the SuperScript III Platinum One-Step qRT-PCR kit (Invitrogen, Waltham, Mass., USA) in each of which specific H5 and H7 primers and probes were inserted, respectively, but they had the same thermal profile: 30 min for 50 °C, 2 min for 95 °C, and 40 of 10 s for 95 °C, 30 s for 54 °C, and 10 s for 72 °C. Influenza-A-positive samples that were negative for PCR subtypes H5 or H7 were subjected to complete sequencing by next-generation sequencing (NGS).

Finally, the thermal profile for the detection of NDV [31] was 50 °C for 30 min, 95 °C for 15 min, and 40 of 94 °C for 10 s, 52 °C for 30 s, and 72 °C for 10 s. Positive samples were subjected to reverse transcriptase polymerase chain reaction (RT-PCR) targeting the fusion (F) protein gene [32] with One-Step RT PCR Kit (Qiagen, Hilden, Germany) under the following amplification steps: 50 °C for 30 min, 95 °C for 15 min and 45 cycles of 94 °C for 30 s, 64 °C for 30 s, and 72 °C for 40 s and elongation at 72 °C for 5 min. The segments obtained at 532 bp were sequenced by the Sanger method. We performed phylogenetic and molecular analyses of the variable region of the F gene (42–421 nt), including the cleavage site, as standard tool for molecular characterization of NDV sequences and for determination of the potential virulence of isolates.

## 3. Results

Molecular analyses performed on wild bird samples in order to detect arboviruses showed a total prevalence of 4% (CI 95%: 3–5) for WNV, with 27 positive birds, mainly belonging to the order Strigiformes, Accipitriformes, and Passeriformes, and originating from different provinces of Lombardy. Real-time RT-PCR performed on positive samples to distinguish lineage 1 and 2 detected lineage 2 in 23 birds. Four samples from *Phalacrocorax carbo*, *Larus michahellis*, *Pica pica*, and *Tachymarptis melba* could not be identified. All birds were negative for the USUV PCR. Table 2 summarises the WNV-positive results in detail.

Regarding avian influenza, only one sample from *Larus michahellis* was positive, and the same gull was positive for WNV. NGS allows identification of the H13N6 virus. Phylogenetic analyses on virus segments highlighted its reassortant nature. Indeed, the segment PB2 belonged to the American lineage, while segments NA, HA, NP, PB1, M, PA, and NS clustered within the Eurasian lineage (see Table 3).

Finally, 12 samples were positive for NDV PCR, in particular, eight *Columba livia*, three *Streptopelia turtur*, and one *Cygnus olor*, with a total prevalence of 1.7% (CI 95%: 1–3). Similarly to birds positive to WNV, subjects came from different provinces. Blast analysis of NDV sequences showed the highest % identity of the swan-origin sequence (99.38%) to NDV sequences detected in geese in Nigeria [33] belonging to the genotype I. All other Italian NDV sequences showed the highest % identity (from 99.58 to 98.53%) to the NDV strain SD18 of pigeon origin, which belongs to genotype VI.2.1.1.2.2. [34].

Molecular analysis of protein F revealed three different motifs at positions 112–117. The Italian swan-origin NDV sequence showed the 112GKQGRL117 motif typically found in low virulence NDVs [35]. All other sequences originating from pigeons and turtledoves showed two different motifs found in the velogenic NDV strains (112RRQKRF117 and 112RRRKKF117) characterized by multiple basic amino acids at positions 112–116 and a phenyl alanine at position 117.

The main results from the molecular analyses related to the different species are summarized in the Table S1 in the Supplementary Materials.

## 4. Discussion

This study highlights the presence and circulation of influenza viruses, even if limited to only a single H13N6 strain, West Nile, and NDVs in free living avian populations, although with a modest prevalence.

Most of the positive samples are attributable to the WNV, which involves a wide range of species. WNV represents a severe public health concern. In Italy, in recent years, there have been a large number of human cases, with the increasingly frequent manifestation of neuro-invasive forms. The peak was recorded in 2018 with more than 500 cases in humans and an increase in isolation in other susceptible species (equids), and in species involved in the virus life cycle: birds and mosquitoes. In wild birds, 321 specimens tested positive for the virus; of these, 215 were from surveillance of target species (crows, magpies, and jays), while 106 were from passive monitoring of wild animals found dead. The number of cases found in 2019 was significantly lower than that in the previous year, slightly rising by 2020 [36]. Indeed, the 2020 European bulletin reported in Italy 144 confirmed cases of WNV among wild birds; even in Germany and Bulgaria, several cases (28 and 2, respectively) in wild birds were reported [36].

The results of this study confirm positivity in orders characterised by species most susceptible to infection; in particular, Accipitriformes, Charadriiformes, Falconiformes, Passeriformes, and Strigiformes [20,37,38].

Of the 27 positive wild birds, four samples from magpie, cormorant, swift, and gull, respectively, could not be typed, probably because the positivity values were close to the threshold. In these cases, typing is difficult to obtain. Nevertheless, a technical issue making typing impossible cannot be excluded; in fact, samples derived from wildlife rehabilitation centres are usually stored in non-optimal conditions (−20 °C) for quite a long time, and they may have been subjected to several freeze and thawing steps during the preparation process, which may have compromised the virus and affected the quality of the analysis. Apart from the magpie, which is listed as a target species for WNV research in the national control plan, the other three species, whose lineage is unknown, are rarely involved in outbreaks of WNV. Currently, no other positive findings have been reported for the alpine swift, even though it is considered a species potentially able to introduce the virus from sub-Saharan Africa and amplify it, especially in dry areas [13].

Sporadic episodes of WNV have also been reported in cormorants. Recently, a serological survey carried out in Germany showed the presence of antibodies against West Nile virus in this species [39]; moreover, in the Volga delta area the virus was isolated from cormorants and ticks (*Hyalomma marginatum*) associated with them [40]. Of particular interest is the co-infection (West Nile and Influenza viruses) found in a gull in 2020. The presence of West Nile has already been documented in this species, but with an infrequent occurrence [41,42,43]. In Italy, only one other identification of WNV in a gull was reported in 2020; in particular, lineage 2 was detected [36]. Previous studies carried out in Italy in the same study areas, aimed to genetically characterize the WNV-2 isolated strains, by investigating the genetic diversity of sequences obtained in the period 2015–2018, from mosquitoes, birds, horses, and humans [44]. The study demonstrated the complete segregation of Italian sequences from those of other European countries, highlighting the presence of two different clades (A and B). The clade A became extinct in 2013–2014, while clade B gave rise to several sub-clades characterized by different spatial distribution. The segregation from other European sequences, and the continuous persistence of some sub-clades on the territory, suggest the presence of endemic clades, supporting the hypothesis of local overwintering in Italy [42]. It will therefore be interesting to deepen the study of these aspects by comparing present birds’ sequences with those of mosquitoes, horses, and humans from the same areas. These further analyses could confirm, as expected, the endemic nature of clades previously isolated.

With regard to the influenza virus, molecular analysis identified the type H13N6, which was isolated for the first time in a gull in 1977 [45]. This type of virus is classified as having low pathogenicity and seems to be typical of the order Charadriiformes. Indeed, gulls are widely recognised as reservoirs for LPAI viruses; among the several types of AIV, the hemagglutinin subtypes H13 and H16 are rarely detected in other avian groups, suggesting their maintenance almost exclusively within gull populations [46,47], but other avian species (i.e., turkeys and ducks) could be a spill-over host for the H13 type [46]. The present data and previous identification of H13N6 in gulls in Italy [23] suggest a low diffusion of this virus in the resident gulls’ population. However, only a small number of seagull species have been received, which does not provide a complete picture of the circulation of this flu type. The presence of segments belonging to different lineages has already been reported [48,49]. While it is relatively common to find European lineages in North America, not much data is available on the opposite phenomenon. Thus, the American origin of segment PB2 found in gulls is of particular interest. This could be due to the occasional migration of typically American species in Europe, or to those ubiquitous species (e.g., *Larus ridibundus*) that are widespread in both Europe and America, which could act as vector for European populations, favouring the virus exchange [50]. Molecular investigations showed the complete absence of other avian influenza strains, particularly H5 and H7 HPAI. The European Food Safety Authority (EFSA), every year, draws up a report about surveillance for avian influenza in poultry and wild birds. In the 2020 report, two cases of HPAI from wild birds, belonging to passive surveillance, were reported in Italy with a prevalence of 0.1%. The highest percentages of HPAI positive birds found by passive surveillance were in Denmark (32% of samples), Germany (14%), the Netherlands (12%), and Ireland (12%) [51]. Therefore, monitoring in wild species helped to expand surveillance data on influenza virus, even if the limited sampling in this study of reservoir species, particularly water birds, may have greatly reduced the overall amount of useful and available information.

With regard to NDV, molecular analyses identified almost all positives in the order of Columbiformes, particularly in pigeons and turtle doves. Pigeons are considered among the most important reservoirs of NDV [52], and strains of variable virulence have been isolated from wild pigeons and doves [35,53]. These bird species could play a very important role in the spread of NDV and other avian viruses because of their ethological characteristics and the overlap of their habitats with areas usually urbanised or used by human activity, particularly livestock and poultry farming. In recent decades, a great number of outbreaks of virulent NDV in chickens, originating from wild birds in European countries, have been reported [54]. Indeed, in the last decade, several cases of NDV in wild birds, above all from the Southern and Eastern Europe, were reported [55]. In most cases, positive birds were identified as collared and turtle doves, which are the most susceptible species. However other species, rarely reported, were infected (i.e., Accipitridae and Gaviidae) [54]. Interspecies transmission seems to be an important mechanism contributing to the maintenance, propagation, and spread of APMV-1 [56]. However, this aspect should be considered bi-directionally between domestic and wild bird populations, including the potential diffusion of live vaccine strains designed for domestic poultry into wild birds [57,58]. Most APMV-1 recognised in wild birds is avirulent in poultry; however, strains of low virulence are capable of naturally evolving into high virulence, although this has been documented only sporadically [33,54]. In addition to Columbiformes, several free ranging aquatic fowls are considered to be potential carriers of *avulavirus*-1 [59]. The present data highlight the presence of APMV-1 in a mute swan. In this species, isolation of NDV is mainly occasional [60]; however, it should not be underestimated considering the proven pathogenicity of the *avulavirus* from swans in chickens [61]. Although occasional serological studies conducted in Argentina reported a seroprevalence of 35% [59], sero-epidemiological surveys could certainly provide better indications of the true circulation of this virus in waterfowl populations. However, unfortunately, the use in this study of carcasses found dead or delivered from rehabilitation centres did not allow adequate blood samples to be obtained to conduct specific serological investigations.

Finally, all samples were negative for the presence of the USUV. This result seems strange compared to the positives reported in Italy in previous years and in the same biennium. In fact, since 2006, several outbreaks of USUV were recorded in Central–Northern Italy, with massive mortality in susceptible species [62], and, in 2009, the first human case was registered [63]. In recent years, an increase in the number of wild bird cases has been observed. Indeed, the national bulletins on arboviroses recorded 98, 26, and 87 USUV-positive wild birds, respectively, in 2018, 2019, and 2020 [36]. However, the great heterogeneity of the analysed species, in relation to the limited number of analyses on target species, could explain the negative results obtained during this survey.

In conclusion, the data presented in this study highlight the importance of passive monitoring of wildlife, particularly wild birds, for increasing the information derived from surveillance plans implemented at the regional and national levels, involving a higher number of wild species often less represented and considered in such control programs. From this perspective, wildlife rehabilitation centres may enhance and simplify surveillance efforts for avian-related viruses [64,65].

The analyses performed on these species, also supported by data collected from other neighbourhoods countries (see Table S2 in the Supplementary Materials), could provide a mass of information, which allows a better description of the epidemiological characteristics such as the diffusion and trends of diseases of economic and, especially, zoonotic importance.

The observed prevalence detected for three out of the four viral agents considered in this study was very low; however, it is possible that these were underestimated. Many viral diseases develop asymptomatically in wild birds, causing rare phenomena of intense mortality. Therefore, it is possible that the real reservoirs of infections were hardly contacted. Moreover, we must not underestimate the quality of the samples; although we constantly tried to keep them under optimal conditions, in field conditions this is not always completely applicable. Finally, in spite of the large number of species analysed (likely the most widespread), this sampling approach is not exhaustive of the real variety of species present in the territory, thus limiting the achievements of some and even more useful information. Therefore, although it provides important data on the spread of certain pathogens, it is essential that passive monitoring is always accompanied by active epidemiological surveillance, including sero-surveillance, which focuses on target species for the viruses under study.

## Figures and Tables

**Table 1:** Primer and probe sequences used for the PCRs and the corresponding target gene.

Name	Primer-Probe Name	Sequence	Target Gene
WEST NILE IDENTIFICATION [25]	WN-10533-10522	AAGTTGAGTAGACGGTGCTG	NS3 gene
	WN-10625-10606	AGACGGTTCTGAGGGCTTAC	
	WN-10560-10579-PROBE	fam-CTCAACCCCAGGAGGACTGG-bhq1	
WEST NILE TYPING [26,27]	WN-LCV-F1	GTGATCCATGTAAGCCCTCAGAA	NS3 gene
	WN-LCV-R1	GTCTGACATTGGGCTTTGAAGTTA	
	WN-LCV-S1 PROBE	fam-AGGACCCCACATGTT-mgb	
	WN-LCV-S2 PROBE	vic-AGGACCCCACGTGCT-mgb	
USUTU [28]	USUTU F	ACGGCCCAAGCGAACAGAC	NS5 gene
	USUTU R	GGCTTGGGCCGCACCTAA	
	USUTU PROBE	CY5-CGAACTGTTCGTGGAAGG-BHQ3	
INFLUENZA A [29]	INFLU-124 MOD	TGC AAA GAC ACT TTC CAG TCT CTG	M gene
	INFL-M124	TGC AAA AAC ATC TTC AAG TCT CTG	
	INFLU M25	AGA TGA GTC TTC TAA CCG AGG TCG	
	M+64 PROBE	fam TCAGGCCCCCTCAAAGCCGA tamra	
INFLUENZA H5 [30]	H5LH1-F	ACATATGACTACCCACARTATTCAG	HA2 gene for emoagglutinin H 5
	H5RH1-R	AGACCAGCTAYCATGATTGC	
	H5PRO-probe	fam-TCWACAGTGGCGAGTTCCCTAGCA-tamra	
INFLUENZA H7 [30]	H7-LH6H7-FOR	GGCCAGTATTAGAAACAACACCTATGA	HA2 gene for emoagglutinin H 7
	H7-RH4H7-REV	GCCCCGAAGCTAAACCAAAGTAT	
	H7PRO11-PROBE	fam-CCGCTGCTTAGTTTGACTGGGTCAATCT-bhq1	
NDV [31]	NDV-1M+4100	AGTGATGTGCTCGGACCTTC	APMV-1 gene
	NDV-M−4220	CCTGAGGAGAGGCATTTGCTA	
	NDV-M+4169-PROBE	fam-TTCTCTAGCAGTGGGACAGCCTGC-tamra	
NDV [32]	FOP 1	TACACCTCATCCCAGACAGGGTC	F gene
	NDV-FOP2	AGGCAGGGGAAGTGATTTGTGGC	

**Table 2:** Orders and species of wild bird tested positive for WND.

Order	Species	Number	Prevalence	Type
Accipitriformes	*Accipiter nisus*	4	8% (4/50)	Lineage 2
	*Buteo buteo*	1	4.1% (1/24)	Lineage 2
	*Pernis apivorus*	1	9% (1/11)	Lineage 2
Apodiformes	*Tachymarptis melba*	1	33% (1/3)	not typed
Charadriiformes	*Larus michahellis*	1	3.1% (1/32)	not typed
Columbiformes	*Columba palumbus*	1	12.5.% 1/8)	Lineage 2
Falconiformes	*Falco tinnunculus*	4	3.4% (4/118)	Lineage 2
	*Falco subbuteo*	1	16.7% (1/6)	Lineage 2
Passeriformes	*Corvus cornix*	4	11.7% (4/34)	Lineage 2
	*Pica pica*	1	7.1% (1/14)	not typed
	*Turdus merula*	1	4.1% (1/24)	Lineage 2
Suliformes	*Phalacrocorax carbo*	1	4.3% (1/23)	not typed
Strigiformes	*Athene noctua*	3	4.9% (3/61)	Lineage 2
	*Otus scops*	1	9.1% (1/11)	Lineage 2
	*Strix aluco*	2	10.5% (2/19)	Lineage 2

**Table 3:** Analyses of virus segments belonging to Eurasian (Eu) and American (Am) lineages. Sequences with the highest % of identity are reported.

Segment	NA	HA	NP	PB2	PB1	M	PA	NS
Lineage	Eu	Eu	Eu	Am	Eu	Eu	Eu	Eu
% Identity	96%	96%	98%	97%	98%	99%	97%	98%
Reference sequences	KX978407.1 KX978025.1	MF682848.1 CY185497.1	MF461189.1 MF146988.1	MF461185.1 MH764127.1	MF145750.1 KX979824.1	MK192343.1 MF694084.1	MF148015.1 MF147897.1	MF694157.1 MF694139.1

## Data Availability

Data generated or analysed during this study are included in the published article.

## Supplementary Materials

The following are available online at https://www.mdpi.com/article/10.3390/microorganisms9091957/s1, Table S1: Orders and species of wild bird analysed during the years 2019–2020 in Lombardy and summary of the main molecular results; Table S2: Situation reported in European countries in recent years. Registered percentages refer to molecular analyses carried out on birds’ tissue. In some cases, values refer to serological surveys (in brackets). NF: data not found. In regard to the study period, data about NDV in wild birds are not available.
