# Supplementary material for: Survey on the Presence of Viruses of Economic and Zoonotic Importance in Avifauna in Northern Italy
Source: Microorganisms. 2021 Sep 15;9(9):1957. doi: 10.3390/microorganisms9091957 (PMC8471648; doi:10.3390/microorganisms9091957)
Supplement: Supplementary file 1 [file microorganisms-09-01957-s001.zip › microorganisms-1342023-SI.pdf]

Order	Species	East Area	West Area	Total	2019	2020	West Nile	Usutu	Influenza A	NDV
Accipitriformes	*Accipiter gentilis*	1	2	3	1	2	neg	neg	neg	neg
	Accipiter nisus	42	8	50	25	25	4 POS LIN 2	neg	neg	neg
	Buteo buteo	17	7	24	11	13	1 POS LIN 2	neg	neg	neg
	Circus aeruginosus	1	0	1	/	1	neg	neg	neg	neg
	Milvus migrans	1	1	2	1	1	neg	neg	neg	neg
	Pernis apivorus	8	3	11	4	7	1 POS LIN 2	neg	neg	neg
Anseriformes	*Anas platyrhynchos*	3	15	18	13	5	neg	neg	neg	neg
	Anser anser	0	1	1	/	1	neg	neg	neg	neg
	Aythya fuligula	1	0	1	/	1	neg	neg	neg	neg
	Cygnus	12	4	16	13	3	neg	neg	neg	1 POS
Apodiformes	*Apus apus*	2	12	14	14	/	neg	neg	neg	neg
	Tachymarptis melba	1	2	3	2	1	1POS WNV (not typed)	neg	neg	neg
Bucerotiformes	*Upupa epops*	1	4	5	2	3	neg	neg	neg	neg
Caprimulgoformes	*Caprimulgus europaeus*	2	0	2	/	2	neg	neg	neg	neg
Charadiiformes	*Larus*	19	13	32	14	18	1 POS WNV (not typed)	neg	1 POS H13N6	neg
	Scolopax rusticola	1	0	1	/	1	neg	neg	neg	neg
Ciconiiformes	*Ciconia ciconia*	0	1	1	1	/	neg	neg	neg	neg
Columbiformes	*Columba livia*	20	4	24	15	9	neg	neg	neg	8 POS
	Columba palumbus	2	6	8	4	4	1 POS LIN 2	neg	neg	neg
	Streptopelia turtur	8	8	16	11	5	neg	neg	neg	4 POS
Coraciiformes	*Alcedo atthis*	1	3	4	2	2	neg	neg	neg	neg
Cuculiformes	*Cuculus canorus*	0	2	2	1	1	neg	neg	neg	neg
Falconiformes	*Falco columbarius*	1	0	1	1	/	neg	neg	neg	neg
	Falco peregrinus	6	0	6	3	3	neg	neg	neg	neg
	Falco subbuteo	4	2	6	3	3	1 POS LIN 2	neg	neg	neg
	Falco tinnunculus	86	32	118	58	60	4 POS LIN 2	neg	neg	neg
Galliformes	*Coturnix coturnix*	2	0	2	/	2	neg	neg	neg	neg

	Perdix perdix	0	1	1	1	/	neg	neg	neg	neg
	Phasianus colchicus	1	0	1	1	/	neg	neg	neg	neg
Gruiformes	*Fulica atra*	0	1	1	1	/	neg	neg	neg	neg
	Gallinula chloropus	2	2	4	1	3	neg	neg	neg	neg
	Rallus acquaticus	1	0	1	/	1	neg	neg	neg	neg
Passeriformes	*Anthus pratensis*	1	0	1	1	/	neg	neg	neg	neg
	Carduelis cardeulis	1	4	5	2	3	neg	neg	neg	neg
	Chloris chloris	1	0	1	/	1	neg	neg	neg	neg
	Coloeus monedula	1	0	1	1	/	neg	neg	neg	neg
	Corvus corax	1	0	1	1	/	neg	neg	neg	neg
	Corvus cornix	5	29	34	25	9	3 POS LIN 2	neg	neg	neg
	Delichon urbicum	0	8	8	4	4	neg	neg	neg	neg
	Emberiza schoeniculus	1	0	1	1	/	neg	neg	neg	neg
	Erithacus rubecula	0	1	1	1	/	neg	neg	neg	neg
	Ficedula hypoleuca	0	1	1	/	1	neg	neg	neg	neg
	Fringilla coelebs	0	2	2	2	/	neg	neg	neg	neg
	Garrulus glandarius	0	4	4	2	2	neg	neg	neg	neg
	Hirundo rustica	0	5	5	5	/	neg	neg	neg	neg
	Oriolus oriolus	0	1	1	1	/	neg	neg	neg	neg
	Parus major	0	1	1	1	/	neg	neg	neg	neg
	Passer domesticus	0	2	2	2	/	neg	neg	neg	neg
	Passer italiae	2	4	6	2	4	neg	neg	neg	neg
	Passer montanus	0	2	2	2	/	neg	neg	neg	neg
	Phoenicurus ochruros	0	1	1	1	/	neg	neg	neg	neg
	Pica pica	1	13	14	7	7	1 POS WNV (not typed)	neg	neg	neg
	Serinus serinus	0	1	1	/	1	neg	neg	neg	neg
	Sturnus vulgaris	2	0	2	1	1	neg	neg	neg	neg
	Sylvia atricapilla	1	0	1	1	/	neg	neg	neg	neg
	Turdus merula	5	19	24	23	1	1 POS LIN 2	neg	neg	neg

Pelecaniformes	*Ardea cinerea*	14	2	16	11	5	neg	neg	neg	neg
	Botaurus stellaris	1	0	1	/	1	neg	neg	neg	neg
	Bubulucus ibis	5	3	8	4	4	neg	neg	neg	neg
	Egretta garzetta	1	4	5	1	4	neg	neg	neg	neg
	Geronticus eremita	0	1	1	/	1	neg	neg	neg	neg
	Nycticorax nycticorax	1	2	3	2	1	neg	neg	neg	neg
	Threskiornis aethiopicus	2	0	2	1	1	neg	neg	neg	neg
Phoenicopteriformes	*Phoenicopterus roseus*	0	1	1	1	/	neg	neg	neg	neg
Piciformes	*Dendrocopos major*	2	2	4	2	2	neg	neg	neg	neg
	Picus viridis	7	6	13	8	5	neg	neg	neg	neg
Podicipediformes	*Podiceps cristatus*	2	0	2	/	2	neg	neg	neg	neg
Strigiformes	*Asio flammeus*	2	0	2	2	/	neg	neg	neg	neg
	Asio otus	5	1	6	2	4	neg	neg	neg	neg
	Athene noctua	39	22	61	31	30	3 POS LIN 2	neg	neg	neg
	Otus scops	6	5	11	4	7	1 POS lIN 2	neg	neg	neg
	Strix aluco	14	5	19	9	10	3 POS LIN 2	neg	neg	neg
	Tyto alba	1	0	1	/	1	neg	neg	neg	neg
Suliformes	*Phalacrocorax carbo*	23	0	23	5	18	1 POS WNV (not typed)	neg	neg	neg
	**Total**	**393**	**286**	**679**	**372**	**307**				

Table S1. Orders and species of wild bird analysed during the years 2019-2020 in Lombardy and summary of the main molecular results.

Table S2. Situation reported in European countries in recent years. Registered percentages refer to molecular analyses carried out on birds' tissue. In some cases, values refer to serological surveys (in brackets). NF: data not found. In regard to the study period, data about NDV in wild birds are not available.

Country	WNV (%)	USUV (%)	AIV (%) [51]
Austria [66]	NF	77.9	NF
Belgium [67]	NF	27.2	6.5
Czech Republic [67]	NF	36	NF
Croatia [68-69]	0.2	0.2	NF
Denmark	NF	NF	31.9
France	NF	NF	2.2
Germany [39]	0	3.5 (PCR on sera)	14.3
Hungary [66,70]	9.3	2.3	0.2
Ireland	NF	NF	11.5
Netherlands [71]	NF	71.5	12.4
Norway	NF	NF	3.9
Poland [72]	35.7	7.14 (sera)	5.2
Serbia [67,73]	11.61	0	NF
Slovenia [74]	4.7 (sera)	NF	2.2
Spain [75-76]	18.23 (sera)	2.1 (sera)	0.2
Switzerland [67]	NF	5.3 (sera)	NF
Sweden	NF	NF	1.7
United Kingdom [67]	NF	0	7.9
Italy			
Emilia Romagna [23,77]	6.5	4.5	0
Lombardy [78]	0.9	NF	NF
Piedmont [79]	0.9	0.3	0
Veneto [80]	4.5	NF	0
Friuli-Venezia-Giulia [80]	12	NF	0

Reference for table S2:

[23] National surveillance plan for avian influenza – 2021: https://www.izsvenezie.it/documenti/temi/influenza-aviaria//piani-sorveglianza/piano-nazionale-influenza-aviaria-2021.pdf (accessed on 25/07/2021).

[39] Michel, F.; Sieg, M.; Fischer, D.; Keller, M.; Eiden, M.; Reuschel, M.; Schmidt, V.; Schwehn, R.; Rinder, M.; Urbaniak, S.; Müller, K.; Schmoock, M.; Lühken, R.; Wysocki, P.; Fast, C.; Lierz, M.; Korbel, R.; Vahlenkamp, T.W.; Groschup, M.H.; Ziegler, U. Evidence for West Nile virus and Usutu virus infections in wild and resident birds in Germany, 2017 and 2018. *Viruses* **2019**, *11(7)*, 674.

[51] Annual Report on surveillance for avian influenza in poultry and wild birds in Member States of the European Union in 2020: https://www.efsa.europa.eu/sites/default/files/2021-07/9985.pdf (Accessed on 30/08/2021).

[66] Weidinger, P.; Kolodziejek, J.; Bakonyi, T.; Brunthaler, R.; Erdélyi, K.; Weissenböck, H.; Nowotny, N. Different dynamics of Usutu virus infections in Austria and Hungary, 2017–2018. *Transbound. Emerg. Dis*. **2020**, *67*(1), 298-307
[67] Vilibic-Cavlek, T., Petrovic, T., Savic, V., Barbic, L., Tabain, I., Stevanovic, V., Klobucar, A.; Mrzljak, A.; Ilic, M.; Bogdanic, M.; Benvin, I.; Santini, M.; Capak, K.; Monaco, F.; Monaco, F.; Savini, G. Epidemiology of Usutu Virus: The European Scenario. *Pathogens*, **2020**, *9(9*), 699.
[68] Vilibic-Cavlek, T.; Savic, V.; Sabadi, D.; Peric, L.; Barbic, L.; Klobucar, A.; Miklausic, B.; Tabain, I.; Santini, M.; Vucelja, M.; Dvorski, E.; Butigan, T.; Kolaric-Sviben, G.; Potocnik-Hunjadi, P.; Balenovic, M.; Bogdanic, M.; Andric, Z.; Stevanovic, V.; Capak, K.; Balicevic, M.; Listes E.; Savini, G. Prevalence and molecular epidemiology of West Nile and Usutu virus infections in Croatia in the 'One health'context, 2018. *Transbound. Emerg. Dis.* **2019**, *66(5),* 1946-1957.
[69] Vilibic-Cavlek, T., Savic, V., Petrovic, T., Toplak, I., Barbic, L., Petric, D., Tabain, I.; Hrnjakovic-Cvjetkovic, I.; Bogdanic, M.; Klobucar, A.; Mrzljak, A.; Stevanovic, V.; Dinjar-Kujundzic, P.; Radmanic, L.; Monaco, F.; Listes, E.; Savini, G. Emerging trends in the epidemiology of West Nile and Usutu virus infections in Southern Europe. *Front. vet. sci*. **2019,** *6*, 437.
[70] Zana, B.; Erdélyi, K.; Nagy, A.; Mezei, E.; Nagy, O.; Takács, M.; Bakonyi, T.; Forgách, P.; Korbacska-Kutasi, O.; Fehér, O.; Malik, P.; Ursu, K.; Kertész, P.; Kepner, A.; Martina, M.; Süli, T.; Lanszki, Z.; Tóth, G.E.; Kuczmog, A.; Somogyi, B.; Jakab, F; Kemenesi, G. Multi-approach investigation regarding the West Nile virus situation in Hungary, 2018. *Viruses*, **2020**, *12*(1), 123.
[71] Munnink, B. B. O., Münger, E., Nieuwenhuijse, D. F., Kohl, R., Van Der Linden, A., Schapendonk, C. M. E., Van der Jeugd, H.; Kik, M.; J. M. Rijks, J.M.; Reusken, M.E.B.C. Koopmans, M. Genomic monitoring to understand the emergence and spread of Usutu virus in the Netherlands, 2016–2018. *Sci. rep*. **2020**, *10(1*), 1-10
[72] Bażanów, B., Jansen van Vuren, P., Szymański, P., Stygar, D., Frącka, A., Twardoń, J., Kozdrowski, R.; Pawęska, J. T. A survey on West Nile and Usutu viruses in horses and birds in Poland. Viruses **2018**, 10(2), 87.
[73] Petrović, T., Šekler, M., Petrić, D., Lazić, S., Debeljak, Z., Vidanović, D., Ignjatović Ćupina, A.; Lazić, G.; Lupulović, D.; Kolarević, M.; Plavšić, B. Methodology and results of integrated WNV surveillance programmes in Serbia. *PLoS One*. **2018**, *13*(4), e0195439.
[74] Račnik ,J.; Trilar, T.; Jelovšek, M.; Zadravec, M.; Slavec, B.; Zorman Rojs, O. West Nile virus in birds in Slovenia. In: Madić J, editor. *Book of Abstracts. Symposium Epidemiological and Clinical Features of West Nile virus in Croatia and Neighboring Countries*. Zagreb **2012,** 9–10.
[75] Bravo-Barriga, D., Aguilera-Sepúlveda, P., Guerrero-Carvajal, F., Llorente, F., Reina, D., Pérez-Martín, J. E., Jiménez-Clavero, M.A.; Frontera, E. West Nile and Usutu virus infections in wild birds admitted to rehabilitation centres in Extremadura, western Spain, 2017–2019. *Vet. Microbiol.* **2021**, *255*, 109020.
[76] Cano-Terriza, D.; Guerra, R.; Lecollinet, S.; Cerdà-Cuéllar, M.; Cabezón, O.; Almería, S.; García-Bocanegra, I. Epidemiological survey of zoonotic pathogens in feral pigeons (*Columba livia* var. domestica) and sympatric zoo species in Southern Spain. *Comp immunol microbiol inf dis*, **2015**, *43*, 22-27.
[77] Lauriano, A.; Rossi, A.; Galletti, G.; Casadei, G.; Santi, A.; Rubini, S.; Carra, E.; Lelli D.; Calzolari, M.; Tamba, M. West Nile and Usutu Viruses' Surveillance in Birds of the Province of Ferrara, Italy, from 2015 to 2019. *Viruses*, **2021** 13(7), 1367.
[78] Chiari, M., Prosperi, A., Faccin, F., Avisani, D., Cerioli, M., Zanoni, M., Bertoletti, M., Moreno, A.M.; Bruno, R.; Monaco, F.; Farioli, M., Lelli, D.; Lavazza, A. West Nile virus surveillance in the Lombardy region, northern Italy. *Transbound. Emerg. Dis.* **2015**, *62(4)*, 343-349
[79] Rizzo, F.; Borgni, E.; Ghia, C.; Belvedere, M.; Dondo, A.; Monaco, F.; Giammarino, M.; Orusa, R.; Chiavacci, L.; Mandola, M.L. West Nile and Usutu circulation in wild birds from North-West Italy 2015-2018. *Int J of Infec Dis* **2019**, *79*, 147
[80] Santagiuliana, M.; Cunial, G.; Bonato, P.; Barbujani, M.; Zanolla, M.; Favero, L.; Michelutti, A.; Da Rold, G.; Terregino, C.; Ormelli, S.; Gagliazzo L.; Mulatti, P. West Nile virus in North-Eastern Italy: overview of surveillance activities in 2018. *Int J of Infec Dis* **2019**, *79*, 39
